# Three-dimensional poly-(ε-caprolactone) nanofibrous scaffolds directly promote the cardiomyocyte differentiation of murine-induced pluripotent stem cells through Wnt/β-catenin signaling

**DOI:** 10.1186/s12860-015-0067-3

**Published:** 2015-09-03

**Authors:** Yan Chen, Di Zeng, Lu Ding, Xiao-Li Li, Xiong-Tao Liu, Wen-Ju Li, Ting Wei, Song Yan, Jiang-Hui Xie, Li Wei, Qiang-Sun Zheng

**Affiliations:** Department of Cardiology, Tangdu Hospital, Fourth Military Medical University, 1 Xinsi Road, Xi’an, 710038 China; Department of Emergency, Chinese PLA No.401 Hospital, 22 Minjiang Road, Qingdao, 266071 China; Department of Cardiology, Chinese PLA No.401 Hospital, 22 Minjiang Road, Qingdao, 266071 China

**Keywords:** Poly-(ε-caprolactone), Nanofibrous scaffold, Induced pluripotent stem cell, Cardiomyocyte differentiation

## Abstract

**Background:**

Environmental factors are important for stem cell lineage specification, and increasing evidence indicates that the nanoscale geometry/topography of the extracellular matrix (ECM) directs stem cell fate. Recently, many three-dimensional (3D) biomimetic nanofibrous scaffolds resembling many characteristics of the native ECM have been used in stem cell-based myocardial tissue engineering*.* However, the biophysical role and underlying mechanism of 3D nanofibrous scaffolds in cardiomyocyte differentiation of induced pluripotent stem cells (iPSCs) remain unclear.

**Results:**

Here, we fabricated a 3D poly-(ε-caprolactone) (PCL) nanofibrous scaffold using the electrospinning method and verified its nanotopography and porous structure by scanning electron microscopy. We seeded murine iPSCs (miPSCs) directly on the 3D PCL nanofibrous scaffold and initiated non-directed, spontaneous differentiation using the monolayer method. After the 3D PCL nanofibrous scaffold was gelatin coated, it was suitable for monolayer miPSC cultivation and cardiomyocyte differentiation. At day 15 of differentiation, miPSCs differentiated into functional cardiomyocytes on the 3D PCL nanofibrous scaffold as evidenced by positive immunostaining of cardiac-specific proteins including cardiac troponin T (cTnT) and myosin light chain 2a (MLC2a). In addition, flow cytometric analysis of cTnT-positive cells and cardiac-specific gene and protein expression of cTnT and sarcomeric alpha actinin (α-actinin) demonstrated that the cardiomyocyte differentiation of miPSCs was more efficient on the 3D PCL nanofibrous scaffold than on normal tissue culture plates (TCPs). Furthermore, early inhibition of Wnt/β-catenin signaling by the selective antagonist Dickkopf-1 significantly reduced the activity of Wnt/β-catenin signaling and decreased the cardiomyocyte differentiation of miPSCs cultured on the 3D PCL nanofibrous scaffold, while the early activation of Wnt/β-catenin signaling by CHIR99021 further increased the cardiomyocyte differentiation of miPSCs.

**Conclusion:**

These results indicated that the electrospun 3D PCL nanofibrous scaffolds directly promoted the cardiomyocyte differentiation of miPSCs, which was mediated by the activation of the Wnt/β-catenin signaling during the early period of differentiation. These findings highlighted the biophysical role of 3D nanofibrous scaffolds during the cardiomyocyte differentiation of miPSCs and revealed its underlying mechanism involving Wnt/β-catenin signaling, which will be helpful in guiding future stem cell- and scaffold-based myocardium bioengineering.

**Electronic supplementary material:**

The online version of this article (doi:10.1186/s12860-015-0067-3) contains supplementary material, which is available to authorized users.

## Background

The development of effective and radical treatments for irreversible functional cardiomyocyte (CM) loss and the deterioration of cardiac function, which is usually caused by myocardial infarction (MI), have long been concerns of clinicians and scientists [1]. Stem cell-based tissue engineering technology, which differentiates stem cells into CMs and regenerates new functional myocardium, offers a novel and feasible approach for the treatment of MI [2, 3]. For cardiac tissue engineering, an ideal functional cardiac patch that could replace the damaged native myocardium should consist of autologous CMs and biomimetic scaffolds [4].

Induced pluripotent stem cells (iPSCs) [5, 6] are characterized by infinite self-renewal and pluripotent differentiation capacities resembling those of embryonic stem cells (ESCs). More importantly, iPSCs can be reprogrammed from patient-specific somatic cells [7], successfully circumventing the ethical and legal controversies and the potential immune concerns associated with ESC applications. Thus, iPSCs are regarded as the most promising cell source for generating autologous CMs for myocardium regeneration [8].

In addition to stem cells, another key aspect of tissue engineering-based myocardium regeneration is the biomaterial scaffold on which cells are seeded [4]. The scaffold must provide not only space for cell adhesion and survival but also biochemical and biophysical cues to cells through cell-scaffold interactions [9]. These cues are important for directing cell fate and for regulating functional processes, including cell growth, migration, proliferation, and differentiation. Furthermore, the three-dimensional (3D) nanofibrous structure developed by electrospinning technology provides an intriguing microenvironment for stem cell growth and differentiation [10]. Thus, considerable effort has been focused on the application of biomimetic 3D nanofibrous scaffolds to boost cardiac tissue engineering [11].

Most experiments in cardiac tissue engineering studies have used neonatal murine CMs [12] and human/murine ESC/iPSC-derived CMs [13] combined with scaffolds that mimic the composition and/or structure features of the native ECM to some extent. However, limited studies have investigated *de novo* the direct role of artificial fibrous scaffolds in stem cell differentiation into CMs because the large majority of studies administered exogenous differentiation-inducing molecules or chemicals to the artificial fibrous scaffolds [14]. Although the biophysical characteristics of fibrous ECMs such as structure, architecture, and micro- and nanoscale topography are known to be important biophysical signals for regulating cell biology and fate decisions [9, 15, 16], little direct evidence has been reported regarding the CM differentiation process of iPSCs on biomimetic nanofibrous scaffolds, and its underlying mechanisms remain elusive.

The objective of the present study was to investigate the possible effect and mechanism of 3D nanofibrous scaffolds on CM differentiation of miPSCs. For this purpose, we fabricated 3D nanofibrous scaffolds composed of poly-(ε-caprolactone) (PCL) using the electrospinning method. Then, we seeded miPSCs directly on the 3D PCL nanofibrous scaffold without adding any differentiation-inducing molecules using the monolayer culture method. We hypothesized that the nanoscale topography of the 3D PCL nanofibrous scaffold would play a critical role in CM differentiation of miPSCs and that the 3D PCL nanofibrous scaffold would directly induce CM differentiation from miPSCs through monolayer cultivation and non-directed spontaneous differentiation *in vitro*.

## Results

### Characterization of PCL nanofibrous scaffolds

**Fig. 1:**
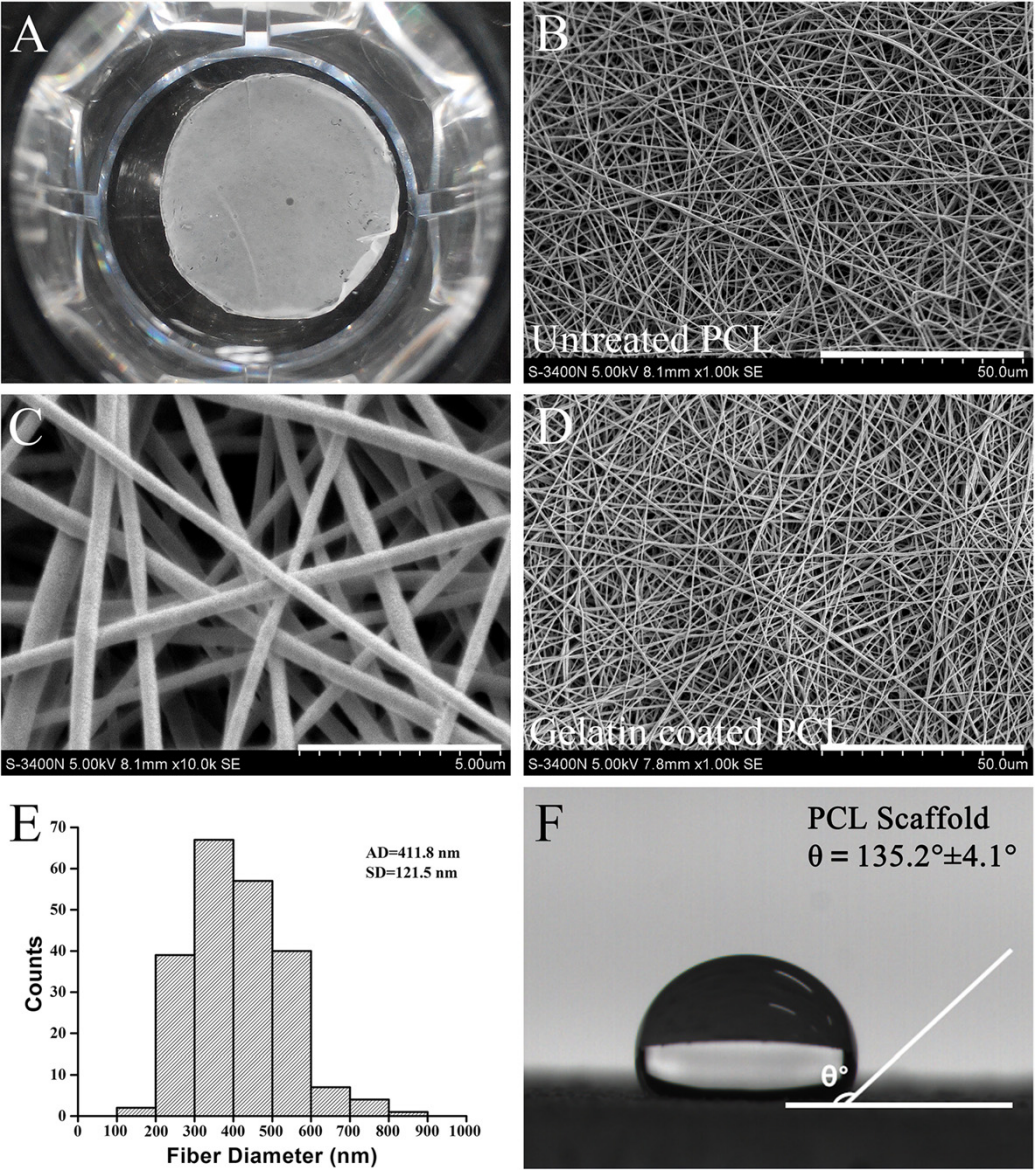
Characterization of poly-(ε-caprolactone) (PCL) nanofibrous scaffolds. **a** A 3D PCL nanofibrous scaffold placed in a 24-well tissue culture plate. **b** Scanning electron microscopy (SEM) images of the electrospun 3D PCL nanofibrous scaffold at 5 000× original magnification; scale bar, 50.0 μm. **c** 10,000× original magnification; scale bar, 5.0 μm. **d** Coated with gelatin, 5000× original magnification; scale bar, 50.0 μm. **e** PCL fiber diameter analysis. AD: average diameter; SD: standard error. *n* = 217 from 6 samples. **f** Water droplet on a 3D PCL nanofibrous scaffold and contact angle (θ) measurement

As shown in Fig. 1, the physical properties of the 3D PCL fibrous scaffolds with and without gelatin coating were characterized. Fig. 1a showed the general morphology of PCL scaffolds in a 24-well polystyrene tissue culture plate (TCP). Scanning electron microscopy (SEM) of the PCL fibrous scaffolds revealed countless smooth fibers without beads, which formed nanoscale structures with interconnected pores (Fig. 1b). The cross arrangement of nanofibers with similar morphology collectively formed a porous 3D structure (Fig. 1c). No obvious change in the surface morphology of the gelatin-coated PCL nanofibers was observed (Fig. 1d). The diameter distribution of the PCL nanofibers was also measured and revealed that most of the fiber diameters ranged from 200 nm to 600 nm; the average diameter was 411.8 nm (Fig. 1e). Specifically, the largest fiber diameter observed was 806.7 nm.

The hydrophilicity of the PCL nanofibrous scaffolds was assessed by measuring the water contact angle, as shown in Fig. 1f. The PCL scaffolds without gelatin coating had much higher contact angles than those of the gelatin-coated PCL scaffolds. The contact angle of uncoated PCL scaffolds was 135.2 ± 4.1 degrees, indicating that the uncoated PCL scaffolds were highly hydrophobic. In contrast, the water contact angle of the gelatin-coated PCL scaffolds was zero, indicating that these scaffolds were extremely hydrophilic. These results revealed that the porous microstructure of the electrospun 3D PCL nanofibrous scaffolds was not altered by gelatin coating and that the hydrophilicity of the PCL scaffolds was significantly improved by gelatin coating.

### miPSC proliferation on 3D PCL nanofibrous scaffolds

**Fig. 2:**
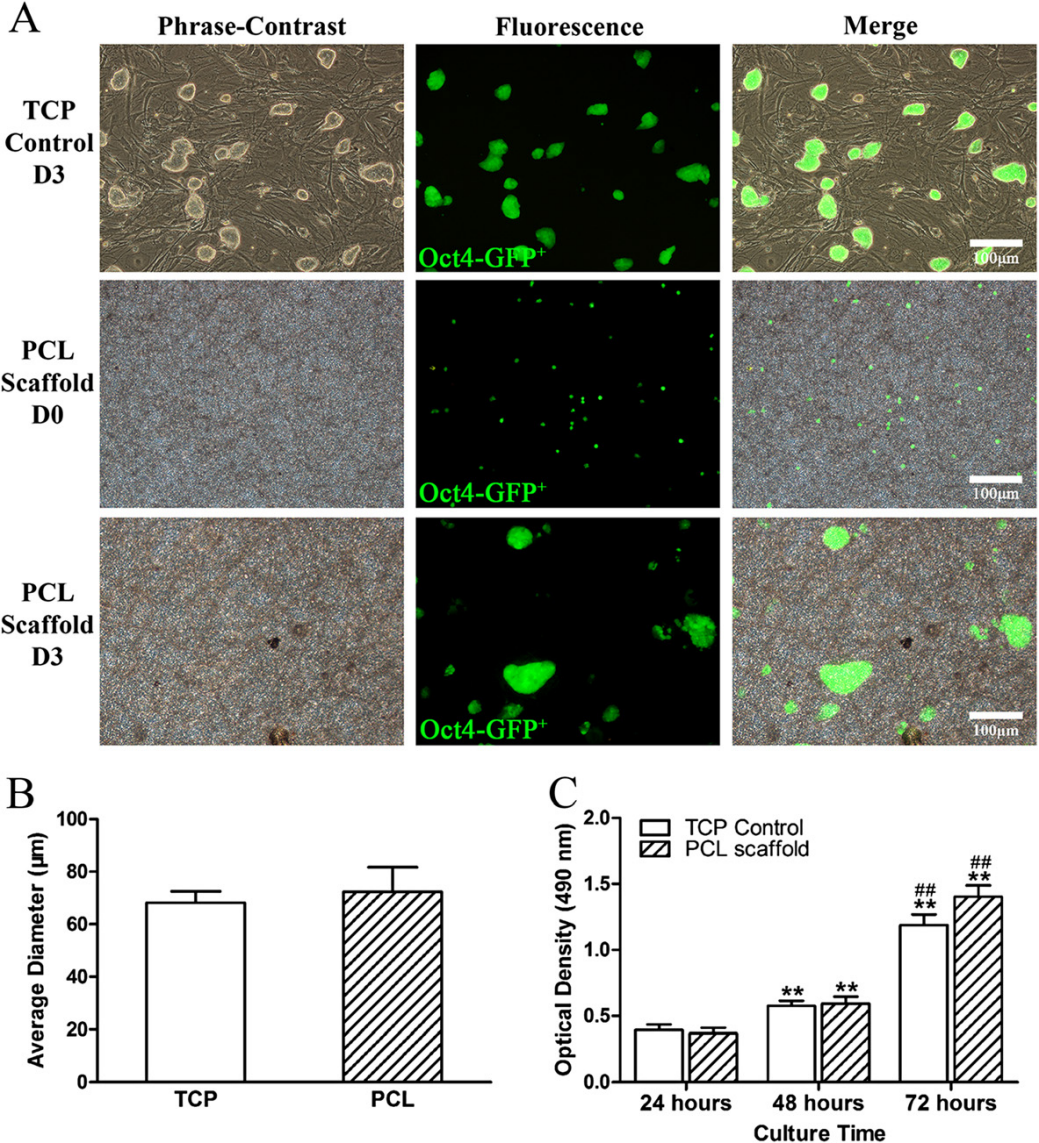
miPSC proliferation on PCL nanofibrous scaffolds. **a** Images of Oct4-GFP^+^ miPSCs cultured on 3D PCL nanofibrous scaffolds and in tissue culture plates (TCPs) with mouse embryonic fibroblast (MEF) feeder layers for the indicated times. **b** Colony diameter analysis. **c** MTT assay for miPSC proliferation on gelatin-coated 3D PCL nanofibrous scaffolds (PCL scaffold) and in gelatin-coated TCPs (TCP Control). All data are presented as the mean ± SE. ^*^Significantly different compared with the TCP control group at 24 h. ^#^Significantly different compared with the PCL scaffold group at 48 h. ^**, ##^ *P* < 0.01. *n* = 5

Next, we evaluated the cytocompatibility of the gelatin-coated PCL scaffolds. GFP^+^ colonies of miPSCs carrying the GFP transgene targeted to the Oct4 locus were observed in the undifferentiated miPSCs (Fig. 2a). At day 0 of co-culture (D0), Oct4-GFP^+^ miPSC colonies were scattered across the scaffold (Fig. 2a, PCL Scaffold D0). At day 3 of co-culture (D3), many compact, dome-like cell colonies were formed by Oct4-GFP^+^ miPSCs on the PCL scaffold (Fig. 2a, PCL Scaffold D3), similar to colonies on the gelatin-coated TCPs (Fig. 2a, TCP Control D3). At day 3 of culture, the average diameter of Oct4-GFP^+^ miPSC colonies on the PCL scaffolds had relatively large variation; however, the average diameter was comparable to that in the TCP control group (Fig. 2b). The mean diameter in the PCL scaffold group was 72.4 ± 9.3 μm, while the mean diameter in the TCP control group was 68.2 ± 4.4 μm (*P* > 0.05).

In addition, the attachment and proliferation of purified miPSCs on the 3D PCL scaffolds were also semi-quantitatively investigated by MTT assay (Fig. 2c). At 24 h of culture, the optical density (OD) values of both groups were comparable to each other (*P* > 0.05). As the culture time increased to 48 h, the mean OD values significantly increased by 46.7 % and 51.1 % in the TCP control and PCL scaffold groups, respectively (**, compared with the TCP control group at 24 h, *P* < 0.01). At 72 h of culture, the OD value in the TCP control group had tripled (3.0-fold), and the OD value in the PCL scaffold group had increased to 3.6-fold (** *P* < 0.01; ^##^ compared with the PCL scaffold group at 48 h, *P* < 0.01). Although the mean OD value in the PCL scaffold group seemed to increase over 72 h, no significant difference was observed compared with the TCP control group at any of the time points (*P* > 0.05). Collectively, these findings suggested that the miPSCs could adhere to and proliferate on 3D PCL nanofibrous scaffolds over time with or without murine embryonic fibroblasts (MEFs).

### Spontaneous cardiac differentiation of monolayer cultured miPSCs on 3D PCL nanofibrous scaffolds

**Fig. 3:**
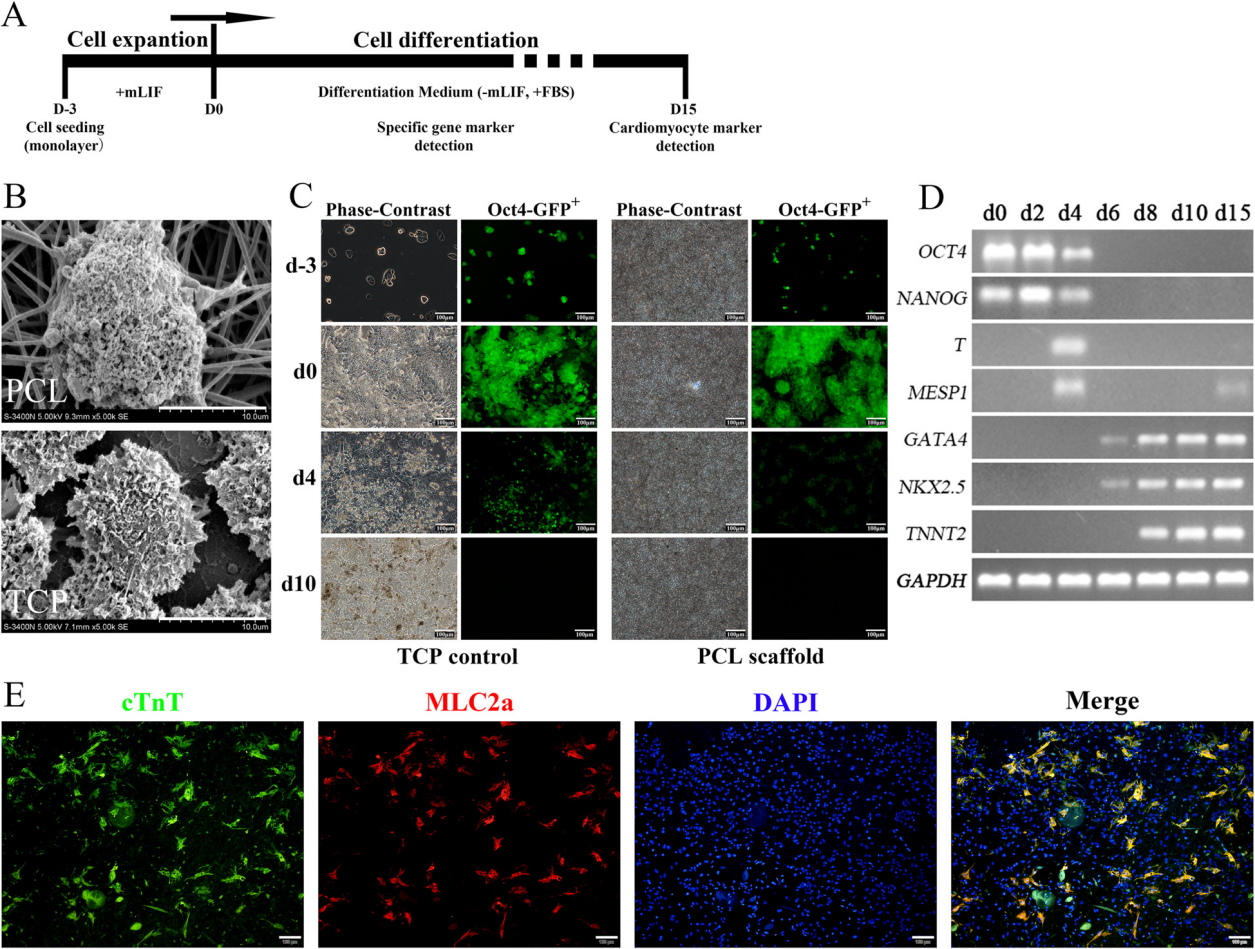
Spontaneous cardiac differentiation of monolayer cultured miPSCs on 3D PCL nanofibrous scaffolds. **a** Schematic of the protocol for the spontaneous CM differentiation of Oct4-GFP^+^ miPSC monolayers cultured without MEF feeder layers *in vitro*. **b** SEM images of the cell morphologies of purified miPSCs on gelatin-coated 3D PCL nanofibrous scaffolds (top) and in gelatin-coated TCPs (bottom) after 1 day of monolayer culture. 5000× magnification; scale bar, 10.0 μm. **c** Images of the spontaneous differentiation of miPSC monolayers cultured on 3D PCL nanofibrous scaffolds and in TCPs at different time points; scale bars, 100 μm. **d** RT-PCR analysis of gene expression of spontaneously differentiated miPSC monolayer cells over 15 days of differentiation on 3D PCL nanofibrous scaffolds. **e** Immunofluorescence images acquired on day 15 of spontaneous differentiation on 3D PCL nanofibrous scaffolds identifying both cTnT and MLC2a with DAPI for nuclei; scale bars, 100 μm

To explore the influence of the nanofibrous topography and porous structure on the CM commitment of miPSCs, the purified Oct4-GFP^+^ miPSCs without MEFs were seeded on the 3D PCL nanofibrous scaffold and in TCPs for CM spontaneous differentiation using the monolayer culture method. The cell culture and differentiation protocol is presented in Fig. 3a. The miPSC morphology, as well as the interactions with the PCL nanofibers or with the surface of the TCPs, was captured by SEM, which revealed the morphological differences between the two groups (Fig. 3b). A more rounded and cilium-like structure was observed with the miPSCs cultured on 3D PCL nanofibrous scaffolds (Fig. 3b, top), while many pseudopodium-like structures with irregular cell edges were observed with the miPSCs cultured in TCPs (Fig. 3b Bottom). Furthermore, dynamic changes in GFP fluorescence and in cell morphology were observed during the spontaneous differentiation period. During differentiation, Oct4-GFP^+^ expression decreased gradually in both groups, which indicated the loss of pluripotency and the progression of differentiation (Fig. 3c). At day 10 of differentiation, floating cells and debris were also found in the media, as shown in images of D10 in Fig. 3c.

To characterize the phenotype of CM differentiation, the expression of a series of marker genes was assessed in miPSCs cultured on PCL scaffolds during the differentiation period. The semiquantitative RT-PCR results showed the rapid concurrent down-regulation of the pluripotent markers *OCT3/4* and *NANOG* during the early period of differentiation, the sequential up-regulation of the `early mesoderm marker gene *BRACHYURY* (*T*) and the basic helix-loop-helix transcription factor mesoderm posterior 1 (*MESP1*), followed by the up-regulation of the cardiac transcription factors GATA-binding protein 4 (*GATA4*) and NK2 homeobox 5 (*NKX2.5*), and finally, the up-regulation of the cardiac-specific structural protein gene cardia troponin T (*TNNT2*) (Fig. 3d). Furthermore, double immunostaining of the cardiac-specific protein markers cTnT and cardiac myosin light chain 2a (MLC2a) at day 15 of differentiation showed that the miPSC-derived cells on 3D PCL nanofibrous scaffolds co-expressed cTnT and MLC2a, indicating the CM phenotype (Fig. 3e). Taken together, these findings demonstrated that the miPSCs cultured on 3D PCL nanofibrous scaffolds using the monolayer method could spontaneously differentiate into functional CMs *in vitro*.

### PCL nanofibrous scaffolds promote the CM differentiation of miPSCs

**Fig. 4:**
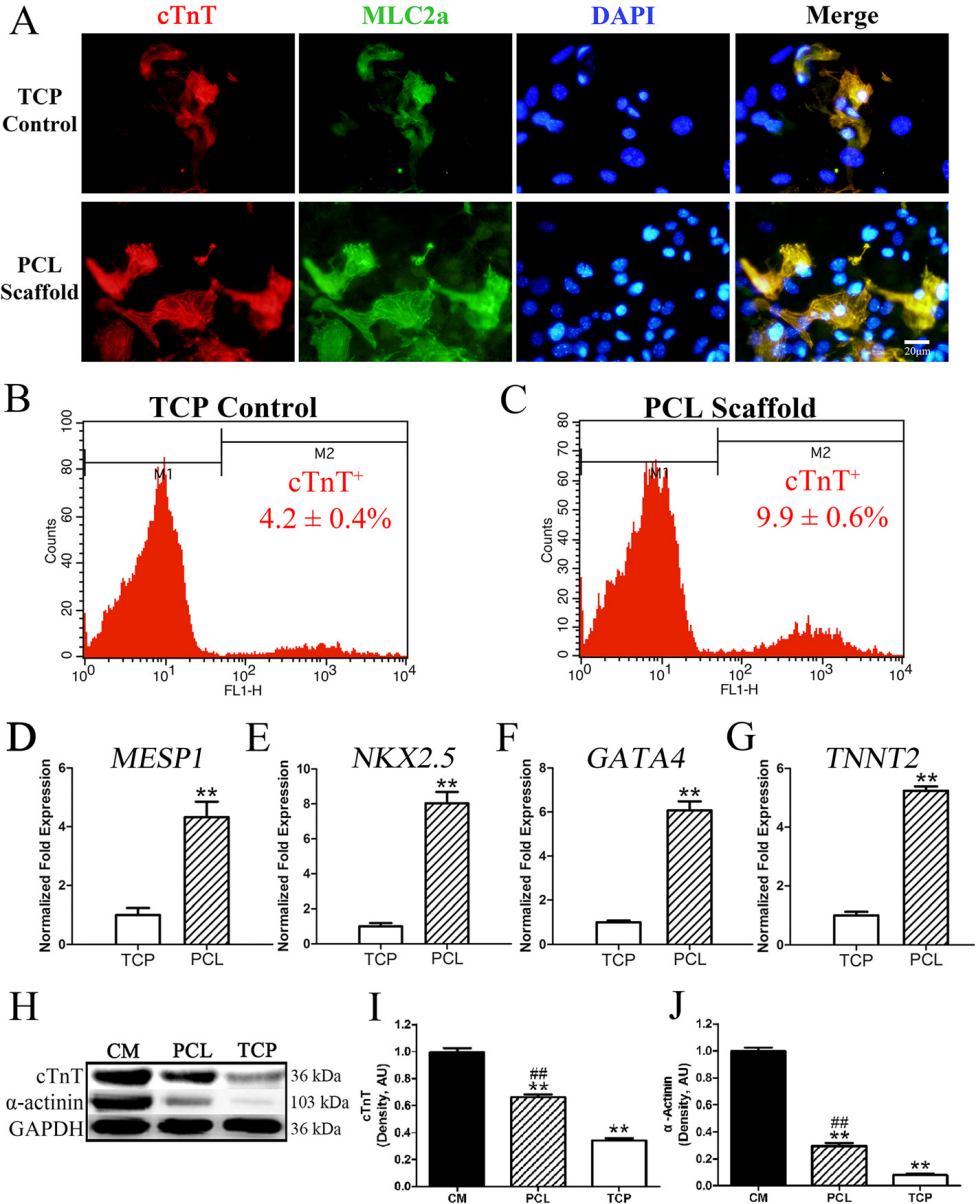
PCL nanofibrous scaffolds promote the cardiomyocyte differentiation of miPSCs. **a** Co-labeling of the spontaneously differentiated miPSC cardiomyocyte (CM) monolayer after 15 days with cTnT and MLC2a antibodies. Scale bar, 20 μm. **b** Flow cytometric analysis of cells from TCP and (**c**) PCL scaffold monolayer culture for cTnT at 15 days differentiation. *n* = 3 independent experiments. **d**–**g** Quantitative RT-PCR analysis of gene expression in the PCL and TCP groups. Total RNA was isolated from cells in TCPs and on 3D PCL nanofibrous scaffolds at 4 days after differentiation for *MESP1* (**d**) analysis and at 10 days after differentiation for *NKX2.5* (**e**), *GATA4* (**f**) and *TNNT2* (**g**) analyses. The expression levels of each gene were normalized to the endogenous control *GAPDH*. The fold changes relative to TCPs from triplicate experiments are presented. **Significantly different between the PCL and TCP groups, **P < 0.01. **h** Representative immunoblots of cTnT, α-actinin and GAPDH in mouse neonatal CMs, miPSC-derived cells on PCL scaffolds and in TCPs. **i** Quantification of cTnT and (**j**) α-actinin protein levels in mouse neonatal CMs, miPSC-derived cells on PCL scaffolds and in TCPs. **Significantly different between the PCL or TCP group *versus* the CM group, ^##^Significantly different between the PCL and TCP groups, **^##^ *P* < 0.01. *n* = 5. All values are presented as the mean ± SE

To verify whether the PCL nanofibrous scaffolds directly promote the CM differentiation of miPSCs, the purified Oct4-GFP^+^ miPSCs were monolayer-seeded in gelatin-coated TCPs as a control, and the same differentiation protocol was used. At day 15 of differentiation, double immunostaining with cTnT and MLC2a revealed well-organized sarcomeric myofilaments and more cTnT and MLC2a staining in miPSC-derived cells cultured on 3D PCL nanofibrous scaffolds compared to those in the TCP control group (Fig. 4a). Moreover, flow cytometric analysis of cTnT-positive cells further confirmed the promoting effect of 3D PCL nanofibrous scaffolds on CM differentiation, as evidenced by a significant increase in cTnT-positive cells in miPSC-derived cells on 3D PCL nanofibrous scaffolds compared to those in the TCP control group (9.9 ± 0.6 % *versus* 4.2 ± 0.4 %, *P* < 0.01; Fig. 4b and c). In addition, quantitative real-time PCR analysis was performed to quantify the differences in the expression of the cardiac mesoderm marker *MESP1*, the early cardiac markers *NKX2.5* and *GATA4* and the late CM marker *TNNT2* between the two groups. The data showed that the expression levels of *MESP1*, *NKX2.5*, *GATA4* and *TNNT2* were significantly increased in miPSC-derived cells on 3D PCL nanofibrous scaffolds compared to those in the TCP control group at D15 (*P* < 0.01). Furthermore, Western blot analysis of α-actinin, an actin-binding protein that plays a critical role in Z-line formation and maintenance, and the cardiac-specific proteins cTnT revealed that the expression levels of cTnT and α-actinin in the PCL group were significantly increased compared to those in the TCP group (^##^, *P* < 0.01; Fig. 4h, i and j). However, the cTnT and α-actinin expression levels in both groups were significantly lower than were those of mouse neonatal CMs (**, *P* < 0.01). Taken together, these results indicated that the 3D PCL nanofibrous scaffold could directly promote the CM differentiation of miPSCs, independent of the administration of exogenous differentiation-inducing molecules or chemicals.

### Wnt/β-catenin signal activation contributes to the promoting effect of 3D PCL nanofibrous scaffolds on the CM differentiation of miPSCs

**Fig. 5:**
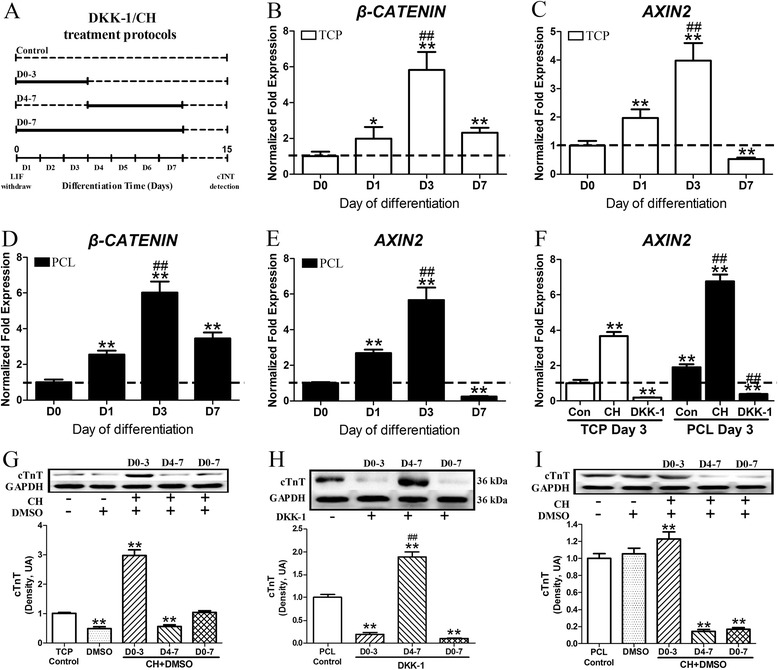
Wnt/β-catenin signaling activation contributes to the 3D PCL nanofibrous scaffold-induced cardiac-directed differentiation of iPSCs. **a** Schematic diagram of the time windows for DKK-1/CH treatment during the differentiation periods. The miPSCs cultured in TCPs or on 3D PCL nanofibrous scaffolds were treated with the canonical Wnt/β-catenin signaling modulators DDK-1 (100 ng/ml) or CH (10 μmol/l) for different periods: D0-3, D4-7 and D0-7. Untreated groups were used as controls. **b**–**f** Quantitative RT-PCR analysis of gene expression of non-treated miPSCs in the PCL and TCP groups at different time points: D0, D1, D3, and D7. **b** *β-CATENIN* and (**c**) *AXIN2* mRNA expression in the TCP group. **d** *β-CATENIN* and (**e**) *AXIN2* mRNA expression in the PCL group. The expression levels of each gene were normalized to the endogenous control *GAPDH*. **Significantly different *versus* D0, ^##^significantly different between D3 and D1, **^##^ *P* < 0.01. *n* = 3 samples. **f** *AXIN2* expression in cells differentiated in TCPs and on 3D PCL nanofibrous scaffolds with or without CH/DKK-1 treatment for 3 days. *GAPDH* was used as endogenous control. The fold changes relative to TCP Control D3 from triplicate experiments are presented. **Significantly different *versus* the TCP Control D3 group, ^##^significantly different *versus* the PCL Control D3 group, **^##^ *P* < 0.01. At the end of the 15 days of differentiation, the cTnT protein levels were determined. Western blot analysis of cTnT expression levels in miPSC-derived cells (**g**) in TCPs treated with CH, **h** on 3D PCL nanofibrous scaffolds treated with DDK-1, and (**i**) on 3D PCL nanofibrous scaffolds treated with CH. **Significantly different between the DKK-1/CH treated groups *versus* the control groups, ^##^significantly different compared with the D0-3 group, **^##^ *P* < 0.01. *n* = 5-7. All results are presented as the mean ± SE. DDK-1: Dickkopf-1, a selective Wnt/β-catenin signaling inhibitor; CH: CHIR99021, a selective Gsk3 inhibitor that activates Wnt/β-catenin signaling

Then, we sought to investigate the underlying mechanism responsible for the promoting effect of the 3D PCL nanofibrous scaffolds on the CM differentiation of miPSCs. *MESP1* and *TNNT2* represent the beginning of the cardiac mesoderm stage of development [17, 18] and the definite appearance of CM, respectively; thus, the early differentiation period was divided into two stages: D0-3 for early cardiac mesoderm genesis and D4-7 for cardiac myocyte commitment. The following period (D8-15) was considered to consist primarily of miPSC-derived CM maturation (Fig. 5a). Increasing evidence has suggested that the Wnt/β-catenin signaling pathway (also known as canonical Wnt signaling) plays a pivotal role in both myocardial development *in vivo* [19, 20] and CM differentiation of multiple iPSC lines *in vitro* [21, 22]. Therefore, we hypothesized that Wnt/β-catenin signaling might be the underlying mechanism involved in the CM differentiation of miPSCs on the 3D PCL nanofibrous scaffolds.

To verify this hypothesis, we investigated the expression of *β-CATENIN* and the Wnt/β-catenin signal target gene *AXIN2* from day 0 to day 7 (D0, D1, D3 and D7) by quantitative real-time PCR analysis. The expression levels of *β-CATENIN* rapidly increased from D0 to D3 and then decreased at D7 in the TCP and PCL groups (Fig. 5b and d). The expression level of *AXIN2*, which positively correlates with the activity of Wnt/β-catenin signaling, had similar changes in both groups (Fig. 5c and e). Importantly, cells on PCL scaffolds had a higher (1.9-fold) expression level of *AXIN2* at D3 compared to that of the TCP group (Fig. 5f; **, *P* < 0.01). These results indicated that the 3D PCL nanofibrous scaffold activated Wnt/β-catenin signaling at the early cardiac mesoderm genesis stage of miPSC differentiation, which might relate to its promoting effect on CM differentiation.

Next, we examined whether the activation of Wnt/β-catenin signaling could promote the CM development of miPSCs. As illustrated in Fig. 5a, the miPSC monolayers in TCPs were treated with the canonical Wnt/β-catenin signaling activator CHIR99021 (CH) [23] at different periods. To exclude the possibility of DMSO, which was used to dissolve CH in the present study, confounding the results of the following experiments, the effect of DMSO on the CM differentiation of miPSCs was also evaluated. Western blot analysis of cTnT protein expression at D15 showed that the solvent DMSO (less than 0.1 % v/v) significantly reduced the level of cTnT expression in TCPs (**, *P* < 0.01). After CH treatment (10 μmol/L, dissolved in DMSO) from D0-3, *AXIN2* mRNA expression was up-regulated 3.6-fold, and cTnT protein expression tripled compared to that of the non-treated control (Fig. 5g; **, *P* < 0.01). Interestingly, CH treatment from D0-7 did not increase cTnT expression and even reduced cTnT expression when the CH treatment was performed during D4-7 (**, *P* < 0.01). These data suggested that the activation of Wnt/β-catenin signaling during the early period (D0-3) could induce the CM differentiation of miPSCs.

To further confirm whether the promoting effect of the 3D PCL nanofibrous scaffolds is mediated by Wnt/β-catenin signaling during the early period, Dickkopf-1 (DDK-1) [23], a selective inhibitor of Wnt/β-catenin signaling, was added to the differentiation medium at different periods (Fig. 5a). For miPSC differentiation on the 3D PCL nanofibrous scaffolds, treatment with DDK-1 (100 ng/ml) during the early period (D0-3) dramatically decreased *AXIN2* expression (Fig. 5f, compared with PCL Control D3; ##, *P* < 0.01) and markedly reduced cTnT expression compared with the non-treated PCL control (Fig. 5h; **, *P* < 0.01). A similar reduction in cTnT expression was also observed when DDK-1 was administered from D0-7 (Fig. 5h; **, *P* < 0.01). However, when DDK-1 was only administered from D4-7, cTnT expression was increased by 88.6 % (**, *P* < 0.01; Fig. 5h; ^##^, *P* < 0.01). These data demonstrated that activation of Wnt/β-catenin signaling during the early period (D0-3) contributes to the promotion of the CM differentiation of miPSCs on 3D PCL nanofibrous scaffolds.

We further investigated whether early activation of Wnt/β-catenin signaling combined with 3D PCL nanofibrous scaffolds could further promote the CM differentiation of miPSCs. As illustrated in Fig. 5f, c, h administration during the early period (D0-3) significantly increased *AXIN2* expression in cells cultured on the PCL scaffold (^##^, *P* < 0.01). As expected, cTnT expression in cells cultured on the PCL scaffold was also elevated by CH treatment during the early period compared to the PCL scaffold alone (Fig. 5i; **, *P* < 0.01). However, the cTnT expression levels were considerably down-regulated when CH was administered from D4-7 and from D0-7 (**, *P* < 0.01). In addition, DMSO alone did not affect cTnT expression in cells cultured on 3D PCL nanofibrous scaffolds (*P* > 0.05). Thus, these data indicated that 3D PCL nanofibrous scaffolds combined with further activation of Wnt/β-catenin signaling during the early period could synergistically promote the CM differentiation of miPSCs using the monolayer culture method, which demonstrated for the first time that the Wnt/β-catenin signaling pathway is involved in the 3D PCL nanofibrous scaffold-induced CM differentiation of miPSCs.

## Discussion

In this study, we investigated the effect of 3D PCL nanofibrous scaffolds on the CM differentiation of miPSCs *in vitro*. The primary findings of this study are as follows. 1) The gelatin-coated 3D PCL nanofibrous scaffolds are suitable for miPSC cultivation and differentiation. 2) The 3D PCL nanofibrous scaffolds are more potent than conventional polyethylene TCPs for promoting the CM differentiation of miPSCs using the monolayer culture method. 3) The activation of Wnt/β-catenin signaling during the early period is involved in the promoting effect of 3D PCL nanofibrous scaffolds on the CM differentiation of miPSCs. Collectively, our studies have revealed a unique CM-promoting effect associated with the interactions between the nanofibers of 3D PCL nanofibrous scaffolds and the intracellular Wnt/β-catenin signaling of miPSCs, which advances our knowledge regarding the important role of the extracellular matrix (ECM) in iPSC-based cardiac regeneration tissue engineering.

PCL is a synthetic high-molecular polymer approved by the US Food and Drug Administration for therapeutic purposes [24]. Furthermore, the electrospinning process provides the opportunity to engineer scaffolds with micro- to nanoscale topography and high porosity similar to the native ECM [25]. Due to their favorable mechanical and biodegradable properties, electrospun 3D PCL nanofibrous scaffolds have been used for stem cell-based tissue engineering [26, 27]. In a previous study, the micro- and the nanoscale structure and porous 3D architectural properties of the human heart ECM were clearly revealed by SEM images [28], and human cardiac ECM slices were used for the CM differentiation of various stem cells such as ESCs, iPSCs and mesenchymal stromal cells (MSCs) [29]. In the present study, the 3D PCL nanofibrous scaffolds fabricated by electrospinning replicated the porous nanostructure of the native human heart ECM, although biochemically they were totally different. Moreover, our PCL nanofibrous scaffolds bore strong resemblance in both fiber diameter and scaffold topography to the PCL scaffolds fabricated in previous reports [30, 31]. We also showed that gelatin coating, a simple method used in a previous report [32], significantly improved the hydrophilicity of the PCL scaffold without changing its surface morphology and demonstrated that the miPSCs proliferate fast on the 3D PCL nanofibrous scaffolds. More importantly, our PCL nanofibrous scaffolds successfully induced the CM differentiation of miPSCs using the monolayer method. Therefore, our findings indicated that the gelatin-coated 3D PCL nanofibrous scaffold might be an ideal artificial 3D substrate for stem cell culture, proliferation and differentiation.

The biophysical characteristics of fibrous ECM, such as its geometry/topography at the nanoscale level and the transmission of biophysical factors to the cell, are important for cell function and cell fate decisions [9, 16]. Recently, Gupta and colleagues [32] transplanted embryoid bodies (EBs) formed by murine ESCs (mESCs) to polymeric scaffolds consisting of polyethylene glycol (PEG), PCL, and carboxylated PCL (CPCL) and found that the PEG-PCL-CPCL composite scaffolds facilitated the differentiation of mESCs towards functional CMs. However, EBs were pretreated with Noggin in their study, which made deciphering the cardiogenesis effect of these scaffolds difficult. To verify the direct effect of our 3D nanofibrous scaffolds on the CM differentiation of miPSCs, we directly seeded miPSCs on the PCL nanofibrous scaffolds or in TCPs and performed the differentiation protocol in the absence of any additional factors or modulators using the monolayer method. In this study, we ensured the individual cell/scaffold adhesion by the monolayer method and excluded the confounding effects of exogenous factors. Therefore, the biophysical signals including the matrix topography, nanoscale fiber, and porous structure of the 3D PCL nanofibrous scaffolds were demonstrated to be important for CM differentiation.

Many reports have already noted that Wnt/β-catenin signaling can potently regulate the cell fate decisions of iPSCs [21]. Some researchers have reported that early and brief exposure to Wnt3a, a canonical Wnt ligand that can activate canonical Wnt/β-catenin signaling, promoted mesoderm formation and CM genesis in hESCs during the first 48 h of differentiation [33]. *AXIN2* is a transcriptional target of β-catenin signaling and is responsible for the down-regulation of β-catenin signaling [23, 34]. Our data revealed that Wnt/β-catenin signaling was up-regulated in cells cultured on the 3D PCL nanofibrous scaffolds during the early stage of miPSC differentiation and that the activation of canonical Wnt/β-catenin signaling during the early stage by the 3D PCL nanofibrous scaffolds contributed to the promotion of the CM differentiation of miPSCs. Our findings support the concept that individual cells can not only respond to soluble molecules but also sense external insoluble biophysical signals primarily through cell adhesion and intercellular junctions [35]. Direct regulation of Wnt signaling by biophysical signals has also been demonstrated in pre-osteoblasts [36]. These biophysical signals can be transduced into intercellular biochemical signals and thus influence cell fate and function [37].

Interestingly, we found that the silencing of canonical Wnt/β-catenin signaling is necessary during the late stage (D4-7) to favor cardiomyogenesis. These findings are consistent with Willems E, *et al.* [38], who found that Wnt inhibition alone after mesoderm formation is sufficient for cardiac induction. Another study also reported that treatment with XAV939, a small molecule inhibitor of Wnt/β-catenin signaling, immediately following the formation of mesoderm progenitor cells promoted CM development in mESCs [39]. Moreover, the dual roles of Wnt/β-catenin signaling during CM differentiation were also reported using multiple iPSCs and ESCs [21]. Additionally, we observed that the activation of canonical Wnt/β-catenin signaling from D0-3 combined with the 3D PCL scaffold further enhanced the CM differentiation of miPSCs, thus indicating that the 3D PCL nanofibrous scaffolds partially activated canonical Wnt/β-catenin signaling during the early stage to exert its promoting effect on the CM differentiation of miPSCs.

During heart development *in vivo*, many signaling molecules such as Nodal, Activin, bone morphogenetic proteins (*e.g.*, BMP2, BMP4), and Wnt ligands, as well as many different types of cells in addition to CMs, were orchestrated to guarantee appropriate heart formation [40, 41]. Moreover, the miPSCs cultured on scaffolds in our study inevitably differentiated into various cell types of the three germ layers. Therefore, further studies will be required to investigate the role of cell/cell interactions during the CM differentiation of iPSCs on 3D PCL nanofibrous scaffolds. In addition, new techniques are also needed to overcome disadvantages when using PCL nanofibers, such as high fiber variability, slow production speed and poor ability to meet complicated architecture requirements.

## Conclusions

In summary, our study demonstrated that the generated electrospun 3D PCL nanofibrous scaffold is an ideal artificial substrate for both iPSC cultivation and cardiac lineage-specific differentiation. We also revealed that 3D PCL nanofibrous scaffolds could directly promote CM differentiation, which might be mediated by the activation of canonical Wnt/β-catenin signaling during early differentiation. Our findings provide new insight regarding exploring innovative biomimetic scaffolds to direct the cardiac fate of iPSCs efficiently for future myocardial tissue engineering and regenerative medicine.

## Methods

### Ethics statements

All experiments were performed with adherence to the National Institutes of Health Guidelines on the Use of Laboratory Animals and were approved by the Fourth Military Medical University Committee on Animal Care.

### Scaffold fabrication by electrospinning

As shown in the schematic diagram in Additional file 1 (Figure S1), the 3D PCL nanofibrous scaffold was fabricated by electrospinning. Briefly, pellets of PCL with an average molecular weight of 80 kDa (Aldrich, St. Louis, MO, USA) were dissolved in a chloroform/methanol (3:1, v/v) solvent mixture at room temperature (25 ± 1 °C) to obtain a 20 wt % solution. Then, the PCL solution was placed in a 10-ml plastic syringe connected to a blunt stainless steel needle with an inner tip diameter of 0.5 mm. A syringe pump (PHD22/2000, Harvard Apparatus, Holliston, MA, USA) was used to control the flow rate at 2 ml/h. A 20-kV high-voltage power supply and a distance of approximately 15 cm were maintained between the needle and the grounded flat aluminum plate collector (size: 15 × 15 cm^2^) throughout the electrospinning process. Then, the PCL fibrous scaffolds were sterilized under ultraviolet (UV) light for 1 h and washed three times with 70 % ethanol for 5 min each. Finally, the scaffold was pre-coated with gelatin (Sigma-Aldrich, St. Louis, MO, USA) by immersion in a sterilized 0.1 % (1 g/l) gelatin solution overnight.

### Maintenance of miPSC cultures

Transgenic Oct4-GFP^+^ miPSCs (kindly provided by Prof. Duanqing Pei, Chinese Academy of Sciences), which carry the GFP transgene targeted to the Oct4 locus, were established by retroviral transduction of the transcription factors Oct4 and Sox2 into OG2 mouse adult fibroblasts using a pMX-based retroviral vector. Undifferentiated Oct4-GFP^+^ miPSCs were routinely cultured and expanded on mitotically inactivated MEF feeder layers or on gelatin-coated polystyrene TCPs as reported previously [42]. The miPSC culture medium was composed of 85 % knockout high-glucose glutamine-free Dulbecco’s modified Eagle’s medium (DMEM) with sodium pyruvate supplemented with 15 % (vol/vol) knockout serum replacement (KSR), 0.1 mmol/l nonessential amino acid stock, 0.1 mmol/l β-mercaptoethanol, 2 mmol/l GlutaMAX (all from Invitrogen, Carlsbad, CA, USA), and 1000 U/ml murine leukemia inhibitory factor (mLIF; Chemicon, Temecula, CA, USA). The cells were incubated in a humidified incubator at 37 °C in an atmosphere of 5 % CO_2_. The culture medium was changed every other day, and the cells were passaged every 2 to 3 days and plated onto MEF feeder layers again to maintain their pluripotent state.

### Cell seeding on the PCL scaffolds

Before the miPSCs were seeded, the gelatin-coated PCL scaffolds were pre-seeded with a MEF feeder layer, and miPSCs were enzymatically dissociated into single cells using 0.05 % Trypsin-EDTA (Invitrogen). Subsequently, the miPSCs were seeded on the PCL scaffolds or gelatin-coated TCPs with miPSC culture medium at the same density of 5 × 10^4^ cells/ml (1 ml/well). The culture medium was changed every day, and the colony analysis was performed after 3 days of culture.

### Colony diameter analysis

At day 3 of culture, images of the miPSC colonies were acquired using an inverted fluorescence microscope (IX71, Olympus, Tokyo, Japan) connected to a photographic system. The mean diameters of the miPSC colonies seeded on PCL scaffolds and TCPs were measured using ImageJ analysis software and calculated from more than five samples per field in 10 fields.

### MTT cell proliferation assay

The miPSC colonies were first passaged on gelatin-coated TCPs without feeder cells up to 3 times to eliminate residual MEFs. Then, the purified miPSCs were seeded on gelatin-coated PCL scaffolds and gelatin-coated TCPs at the same density of 5 × 10^4^ cells/ml (1 ml/well). After the cells were incubated for 24, 48 or 72 h, MTT assay was performed. For MTT assay, MTT solution was added, and the cells were incubated for an additional 4 h. Then, the solution was aspirated, and dimethyl sulfoxide (DMSO) was added to dissolve the formazan crystals. The final solution was transferred to a 96-well plate, and the absorbance was measured at 490 nm using an xMark™ microplate absorbance spectrophotometer (Bio-Rad Laboratory, Hercules, CA, USA).

### Monolayer culture and miPSC differentiation on PCL scaffolds

Purified Oct4-GFP^+^ miPSCs were dispersed into single cells and were seeded on gelatin-coated PCL scaffolds and TCPs at 5 × 10^5^ cells/cm^2^ in miPSC culture medium without MEF feeder layers. The media were changed daily until the miPSCs attained approximately 90 % confluency. Then, the miPSC culture medium was changed to differentiation medium (defined as day 0), and mLIF was withdrawn. The differentiation medium consisted of 80 % (vol/vol) high-glucose glutamine-free DMEM, 20 % (vol/vol) ES-qualified fetal bovine serum, 0.1 mmol/l nonessential amino acid stock, 0.1 mmol/l β-mercaptoethanol and 2 mmol/l GlutaMAX (all from Invitrogen, Carlsbad, CA, USA). From day 0 until day 15, the miPSCs were maintained in the differentiation medium to allow spontaneous differentiation. The differentiation medium was changed every day.

### SEM and measurement of fiber diameters and water contact angles

The morphology of electrospun PCL fibrous scaffolds with and without gelatin coating and of monolayer miPSCs cultured on scaffolds and plates were assessed by SEM as described previously (33). For SEM of monolayer miPSCs, the samples taken at 24 h following seeding were rinsed with PBS, fixed in 2.5 % glutaraldehyde for 2 h and post-fixed with 1 % osmium tetroxide buffered with 0.1 mol/l sodium cacodylate for 1 h. Then, the fixed samples were dehydrated using a series of ethanol solutions with concentrations from 50 % to 70 %, 80 %, 95 %, and 100 % (v/v). Subsequently, the samples were allowed to air dry in a fume hood at room temperature and then gold sputter-coated. For SEM of electrospun PCL fibrous scaffolds with and without gelatin coating, the samples were directly gold sputter-coated. All samples were observed and imaged using a scanning electron microscope (Hitachi S-3400 N, Tokyo, Japan).

The diameters of the nanofibers were measured from acquired SEM images using ImageJ analysis software (http://rsbweb.nih.gov/ij/download.html) and calculated from more than 10 fields. To evaluate the wettability of the 3D PCL nanofibrous scaffold surface, the water contact angles were measured using the sessile drop method with an Easy Drop Contact Angle Goniometer (Kruss, Hamburg, Germany).

### Immunofluorescence staining

After the cells were allowed to differentiate for 15 days, they were washed with PBS, fixed in 4 % paraformaldehyde for 15 min, permeabilized with 0.5 % Triton-X 100 for 10 min and blocked with 5 % bovine serum albumin (BSA; Sigma-Aldrich, St. Louis, MO, USA) at 37 °C for 30 min. Subsequently, the cells were first incubated with mouse monoclonal anti-cardiac troponin T antibody (cTnT, 1:100; Abcam, Cambridge, MA, USA) for 90 min, followed by staining with goat anti-mouse IgG-FITC (1:400; Santa Cruz Biotechnology, Dallas, TX, USA) for 60 min at room temperature. After the cells were washed three times with PBS to remove the excess staining, they were incubated with rabbit polyclonal anti-myosin light chain (MLC2a, 1:100, Santa Cruz Biotechnology Inc) primary antibody for 90 min at room temperature. This incubation was followed by incubation with goat anti-rabbit IgG-TR (1:400; Santa Cruz) for 60 min at room temperature. After the cells were rinsed with PBS, the nuclei were counterstained with 4’,6-diamidino-2-phenylindole (DAPI; 1:1000; Sigma, St. Louis, MO, USA) for 5 min. The samples were finally imaged using a fluorescence microscope (IX71, Olympus, Tokyo, Japan).

### RT-PCR and quantitative RT-PCR analysis

Total cellular RNA was extracted using TRIzol reagent (Invitrogen, Carlsbad, CA, USA) according to the manufacturer’s instructions, followed by treatment with recombinant DNase I (TaKaRa, Otsu, Shiga, Japan) to remove genomic DNA contamination. RNA (0.1 μg) was reverse transcribed into cDNA using a PrimeScript™ First Strand cDNA Synthesis Kit (TaKaRa). PCR amplification of the cDNA was performed using a standard procedure with Taq DNA Polymerase (TaKaRa). The primer sequences and PCR conditions are listed in Additional file 2 (Table S1). SYBR Green real-time PCR analysis was performed with SYBR Premix Ex Taq™ II (TaKaRa). The PCR products were then subjected to 2 % agarose gel electrophoresis. *GAPDH* was used as an endogenous control. Three replicate samples were processed at each time point. Relative quantification was performed using the ^△△^C_T_ method.

### Western blot analysis

The cell samples collected at day 15 of differentiation were lysed as described previously [21]. The protein concentrations of myocardium samples were determined using a BCA protein assay kit (KeyGen Biotech Co., Ltd., Nanjing, China) in accordance with the manufacturer’s instructions. The quantified protein (100 μg/sample) were separated by SDS-PAGE under denaturing conditions and transferred onto polyvinylidene difluoride (PVDF) membranes (Millipore, Billerica, MA, USA). After the membranes were blocked with 5 % (wt/vol) milk in Tris-buffered saline (TBS: 150 mM NaCl, 50 mM Tris-HCl, pH 7.5) with Tween, they were incubated overnight at 4 °C with primary antibodies against cTnT (rabbit monoclonal anti-mouse cTnT, 1:2000; Abcam, Cambridge, MA, USA), sarcomeric alpha actinin (rabbit monoclonal anti-α-actinin, 1:1000; Abcam, Cambridge, MA, USA) and GAPDH (rabbit monoclonal anti-GAPDH, 1:10,000; Abcam, Cambridge, MA, USA). Then, the membranes were washed to remove excess primary antibody, and the blots were incubated with horseradish peroxidase (HRP)-conjugated secondary antibody at room temperature for 1 h. Binding was detected *via* enhanced chemiluminescence (Millipore, Billerica, MA, USA), and the films were scanned using a ChemiDoc XRS system (Bio-Rad Laboratory, Hercules, CA, USA). The band intensities were quantified using ImageJ software. Three replicates were performed for each time point.

### Flow cytometry

Cells at day 15 of differentiation, which had been detached from the PCL scaffolds or gelatin-coated TCPs using 0.25 % trypsin-EDTA (Invitrogen), were neutralized by FBS, fixed in 1 % paraformaldehyde at 37 °C in a water bath for 10 min, and permeabilized in ice-cold 90 % methanol for 30 min on ice. Then, the cells were washed twice in FACS buffer (PBS with 0.1 % BSA and 0.1 % Triton) and then incubated with primary antibody (rabbit monoclonal anti-mouse cTnT, 1:2000; Abcam, Cambridge, MA, USA) overnight at 4 °C in FACS buffer. Next, the cells were washed twice with 1 ml FACS buffer and then incubated with goat anti-rabbit Alexa 488 (1:1000, Invitrogen) for 30 min in the dark at room temperature. After the cells were washed twice with FACS buffer, they were analyzed using a FACSCaliber flow cytometer (Becton Dickinson, Franklin Lakes, NJ, USA) and CellQuest software.

### Statistical analysis

The data (except fiber counts) are presented as the mean ± standard error of the mean (SEM). The densitometry was analyzed using the Kruskal-Wallis test, followed by a Dunn post hoc test. Other data were subjected to one-way analysis of variance (ANOVA) with Games-Howell post hoc tests for statistical analysis. For all analyses, differences with *P* < 0.05 were considered statistically significant.

### Availability of supporting data

The data sets supporting the results of this article are included within the article and its additional files.

## Additional files

Additional file 1: Figure S1. Schematic diagram of a typical horizontal electrospinning system. (PDF 41 kb) Additional file 2: Table S1. Primers and cycling conditions for RT-PCR. (XLSX 10 kb)

## Abbreviations

ECM: Extracellular matrix
3D: Three-dimensional
PCL: Poly-(ε-caprolactone)
TCP: Tissue culture plate
CM: Cardiomyocyte
MI: Myocardial infarction
ESC: Embryonic stem cell
SEM: Scanning electron microscopy
DDK-1: Dickkopf-1
CH: CHIR99021
IWR: Inhibitor of the canonical Wnt signaling response
