# Supplementary figures and images for: Three-dimensional poly-(ε-caprolactone) nanofibrous scaffolds directly promote the cardiomyocyte differentiation of murine-induced pluripotent stem cells through Wnt/β-catenin signaling
Source: BMC Cell Biol. 2015 Sep 3;16:22. doi: 10.1186/s12860-015-0067-3 (PMC4558999; doi:10.1186/s12860-015-0067-3)

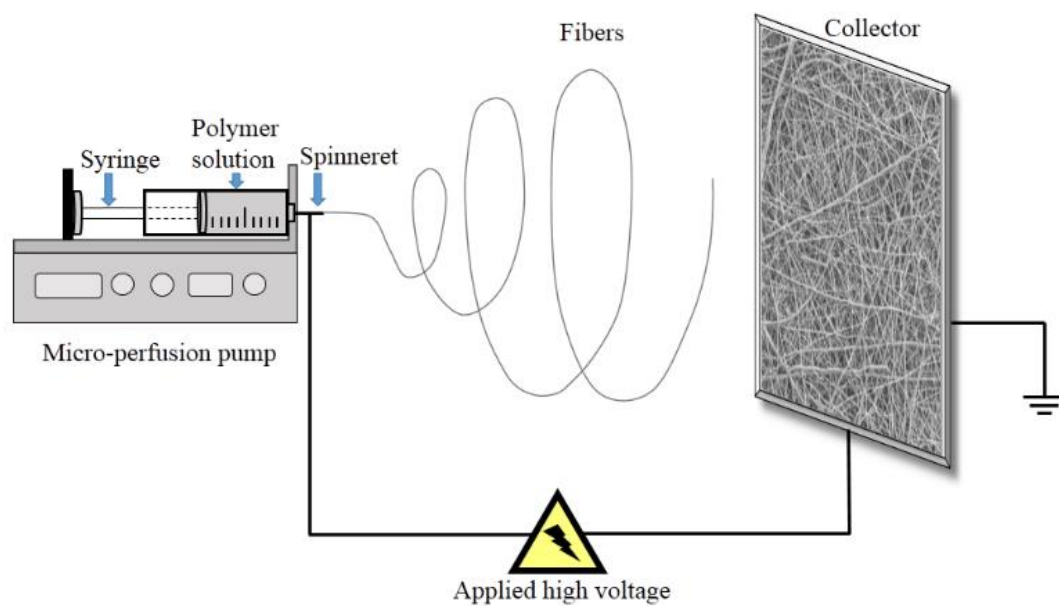

**Figure S1. Schematic diagram of a typical horizontal electrospinning system.**

Supplement: Additional file 1: Figure S1. — Schematic diagram of a typical horizontal electrospinning system. (PDF 41 kb) [file 12860_2015_67_MOESM1_ESM.pdf]
